# Supplementary material for: Order–disorder transition of a rigid cage cation embedded in a cubic perovskite
Source: Nat Commun. 2021 Jun 10;12:3548. doi: 10.1038/s41467-021-23917-z (PMC8192939; doi:10.1038/s41467-021-23917-z)
Supplement: Supplementary file 1 — Supplementary Information [file 41467_2021_23917_MOESM1_ESM.pdf]

Supplementary Information for

Order–Disorder Transition of a Rigid Cage Cation Embedded in a Cubic Perovskite

Zhifang Shi, *Zheng Fang*, *Jingshu Wu*, *Yi Chen and Qixi Mi**

School of Physical Science and Technology, ShanghaiTech University, Shanghai 201210, China.

General 3

Supplementary Figure 1. Synthetic route to 4-azatricyclo[2.2.1.0^{2,6}]heptanium iodide. 3

Synthesis of 4-Azatricyclo[2.2.1.0^{2,6}]heptane 4

Supplementary Figure 2. (a) ^{1}H and (b) ^{13}C NMR spectra of 4-azatricyclo[2.2.1.0^{2,6}]-heptanium iodide in DMSO-d_6. 8

Supplementary Figure 3. ^{1}H NMR spectra of (a) 2-azabicyclo[2.1.1]hexanium and (b) quinuclidinium iodide in DMSO-d_6. 9

Supplementary Figure 4. NMR peak fitting for the α methylene protons of Abh^{+} (blue), Ath^{+} (green), and Q^{+} (red). 10

Supplementary Figure 5. Single-crystal structure of 4-azatricyclo[2.2.1.0^{2,6}]heptanium iodide (AthI) at 150 K. 11

Supplementary Figure 6. Photograph of an AthMn(N_3)$_3$ single crystal next to 0.5 mm rulings. 12

Supplementary Table 1. Crystal data and structural refinement for AthI. 13

Supplementary Table 2. Crystal data and structural refinement for AthMn(N_3)$_3$. 14

Supplementary Table 3. Crystal data and structural refinement for AbhMn(N_3)$_3$. 15

Supplementary Table 4. Crystal data and structural refinement for QMn(N_3)$_3$. 16

Crystallographic Information Files (CIFs) 17

Supplementary References 19

General

Unless otherwise noted, commercial reagents and solvents of analytical grade were used without further purification, and reactions were carried out under a nitrogen atmosphere.

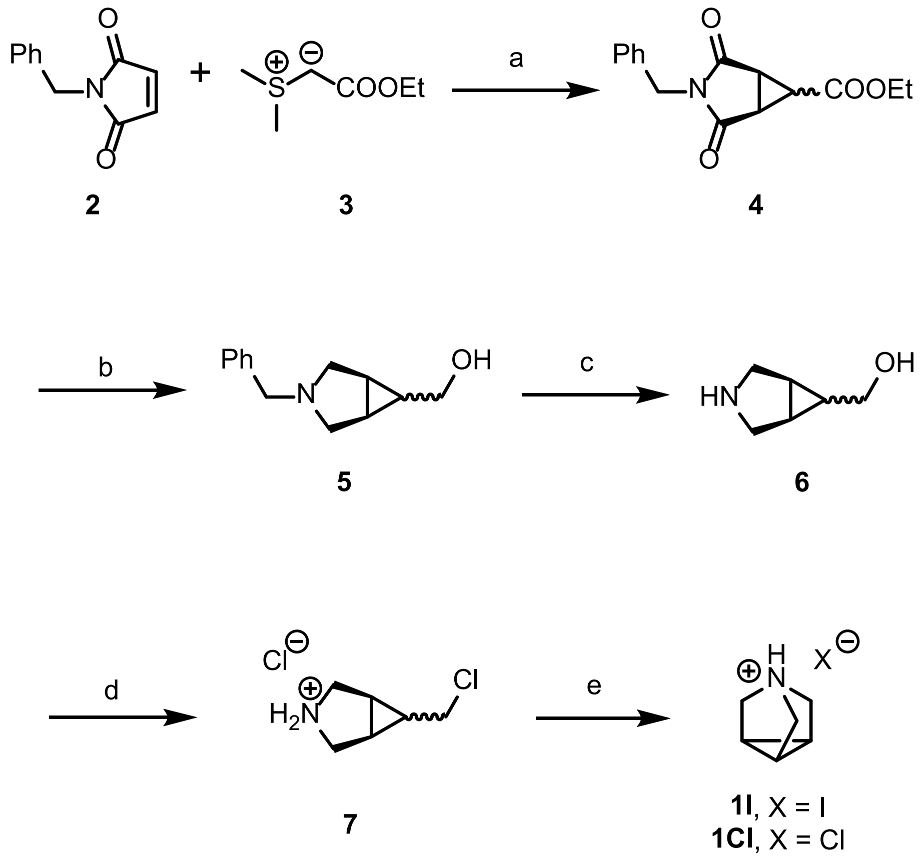

Supplementary Figure 1. Synthetic route to 4-azatricyclo[2.2.1.0^{2,6}]heptanium iodide. Conditions: a) Reflux in 1,2-dichloroethane; b) $LiAlH_4$; c) N_2H_4, Pd/C; d) $SOCl_2$; e) NaOH and then HX.

Synthesis of 4-Azatricyclo[2.2.1.0^{2,6}]heptane

*Cyclopropane imide ester **4** (Ref. 1)*

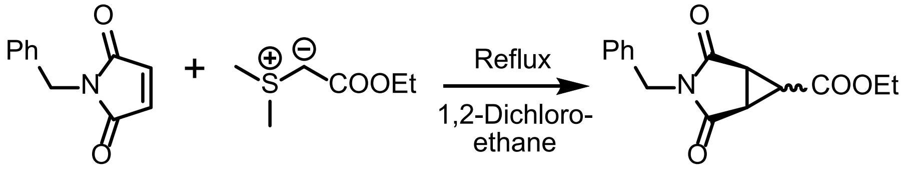

2 **3** **4**

A colorless solution of 4.7 g (25 mmol) *N*-benzylmaleimide (**2**, Bidepharm BD137121) in 60 mL 1,2-dichloroethane was heated to reflux, and added dropwise a solution of 4.3 g (29 mmol) ethyl (dimethylsulfuranylidene)acetate[2] (**3**) in 60 mL 1,2-dichloroethane. The first several drops led to a red intermediate which quickly faded. During 6 h of addition, the intermediate accumulated and the solution turned clear wine red. After addition finished, the reaction was refluxed for another 2 h and then worked up by passing through silica gel and evaporating the solvent. Recrystallization from 15 mL EtOH yielded 5.3 g (19 mmol, 77%) off-white solid **4** as a mixture of two isomers. Preparatory column chromatography (Biotage Isolera One) on silica gel (80 g) eluated by 3:1 petroleum ether–ethyl acetate afforded *exo*-**4** as the first fraction (1.6 g, R_f = 0.47) and *endo*-**4** as the second fraction (2.4 g, R_f = 0.19).

endo-**4**: colorless crystals, m.p. 123.9–124.8 °C. ^{1}H NMR (500 MHz, $CDCl_3$) δ: 7.39 (d, *J* = 7.2 Hz, 2H), 7.35–7.23 (m, 3H), 4.50 (s, 2H), 4.05 (q, *J* = 7.1 Hz, 2H), 2.80 (d, *J* = 8.4 Hz, 2H), 2.54 (t, *J* = 8.4 Hz, 1H), 1.21 (t, *J* = 7.1 Hz, 3H). ^{13}C NMR (126 MHz, $CDCl_3$) δ: 171.22, 167.09, 135.85, 129.03, 128.57, 127.84, 62.09, 42.79, 31.85, 26.75, 14.01. Single-crystal XRD (150 K):

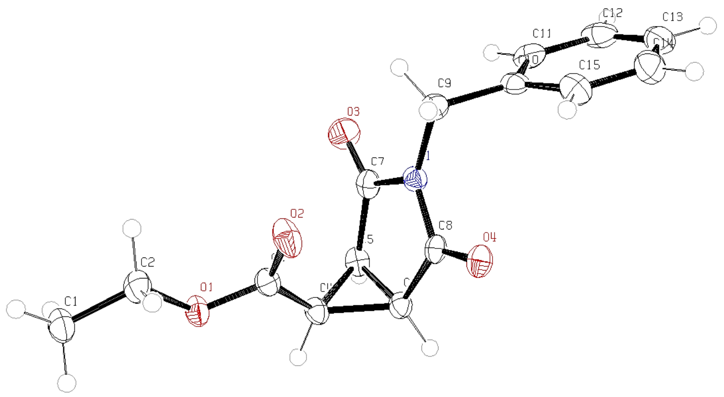

Azabicyclo[3.1.0]hexanemethanol **5**

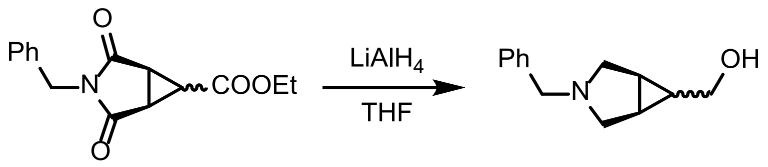

4 **5**

A 500 mL round-bottom flask was charged with 2.3 g (60 mmol) $LiAlH_4$ and 275 mL THF (dried over $LiAlH_4$ and distilled). Under stirring in a cold water bath, a solution of 5.3 g (19 mmol) **4** in 25 mL dry THF was added dropwise and the reaction mixture was refluxed overnight. After quenching by 15 mL saturated NH_4Cl solution, the resulting reaction mixture was filtered through Celite and concentrated to a crude, brown-red oil. Distillation under reduced pressure (102 °C, 17 Pa) yielded 2.5 g (12 mmol, 64%) product **5** as a colorless oil.

endo-**5**: ^{1}H NMR (500 MHz, $CDCl_3$) δ: 7.36–7.28 (m, 2H), 7.28–7.21 (m, 3H), 5.53 (s, 1H), 4.07 (d, J = 4.9 Hz, 2H), 3.64 (s, 2H), 3.15 (d, J = 9.6 Hz, 2H), 2.57(d, J = 9.2 Hz, 2H), 1.58–1.49 (m, 2H), 1.02 (t, J = 8.5, 4.9 Hz, 1H). ^{13}C NMR (126 MHz, $CDCl_3$) δ: 138.14, 128.66, 128.51, 127.32, 59.58, 58.97, 52.98, 22.10, 19.65.

*4-Azatricyclo[2.2.1.0^{2,6}]heptanium iodide **1I***

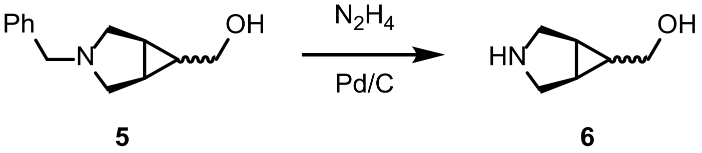

In a 50 mL round-bottom flask was placed 2.5 g (12 mmol) **5**, 20 mL anhydrous ethanol, and 1.5 g 5% Pd/C. Under stirring, a solution of 1.4 mL (25 mmol, 2.0 equiv) 85% hydrazine hydrate in 10 mL ethanol was added dropwise, and the reaction mixture was refluxed for 2 h. The reaction was worked up by filtering through Celite and rotary evaporation to give crude **6** as a nearly colorless oil, 1.4 g (12 mmol, quantitative yield).

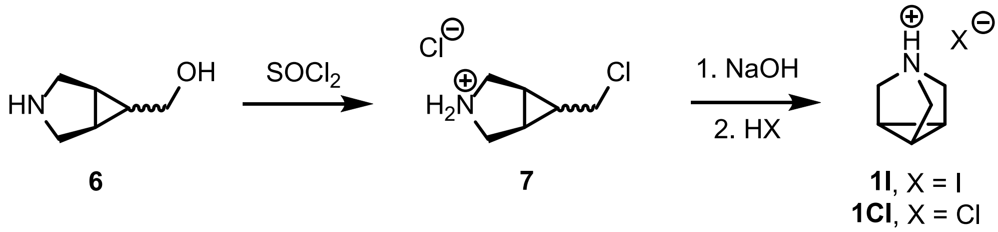

A solution of 1.4 g (12 mmol) **6** in 25 mL 1,4-dioxane was added dropwise to a solution of 1.1 mL $SOCl_2$ (15 mmol) in 5 mL 1,4-dioxane. The reaction mixture was further refluxed for 2 h and turned from orange to brown-black. Evaporation of the solvent and volatiles afforded crude **7** as a brown-black oil, 2.0 g (12 mmol). This crude oil was mixed with 10 mL H_2O and 27 mL 1 M aq. NaOH, stirred at 60 °C overnight, and boiled until the distillate became no longer basic (pH ≤ 9). The collected distillate was titrated by 0.55 M aq. HI and evaporated to dryness, yielding 1.1 g (5.1 mmol, 42% over three steps) crude **1I**. The crude product was recrystallized from 20 mL EtOH and 2 mL H_2O, filtered, and dried to yield 0.70 g (3.2 mmol, 27% separated yield) **1I** as colorless, nonhygroscopic plates. Similarly, neutralization by aq. HCl gave **1Cl** as colorless solid of poor crystallinity but good solubility in polar organic solvents.

1I: ^{1}H NMR (500 MHz, D_2O) δ: 3.17 (s, 6H), 1.87 (s, 3H). ^{1}H NMR (500 MHz, DMSO-d_6)

δ: 10.27 (s, 1H), 3.21 (s, 6H), 1.90 (s, 3H). ^{13}C NMR (126 MHz, D_2O) δ: 56.53, 7.55. ^{13}C NMR (126 MHz, DMSO-d_6) δ: 56.08, 8.21. HRMS (ESI, *m/z*): $[C_6H_{10}N]^+$ experimental 96.0811, predicted 96.0808.

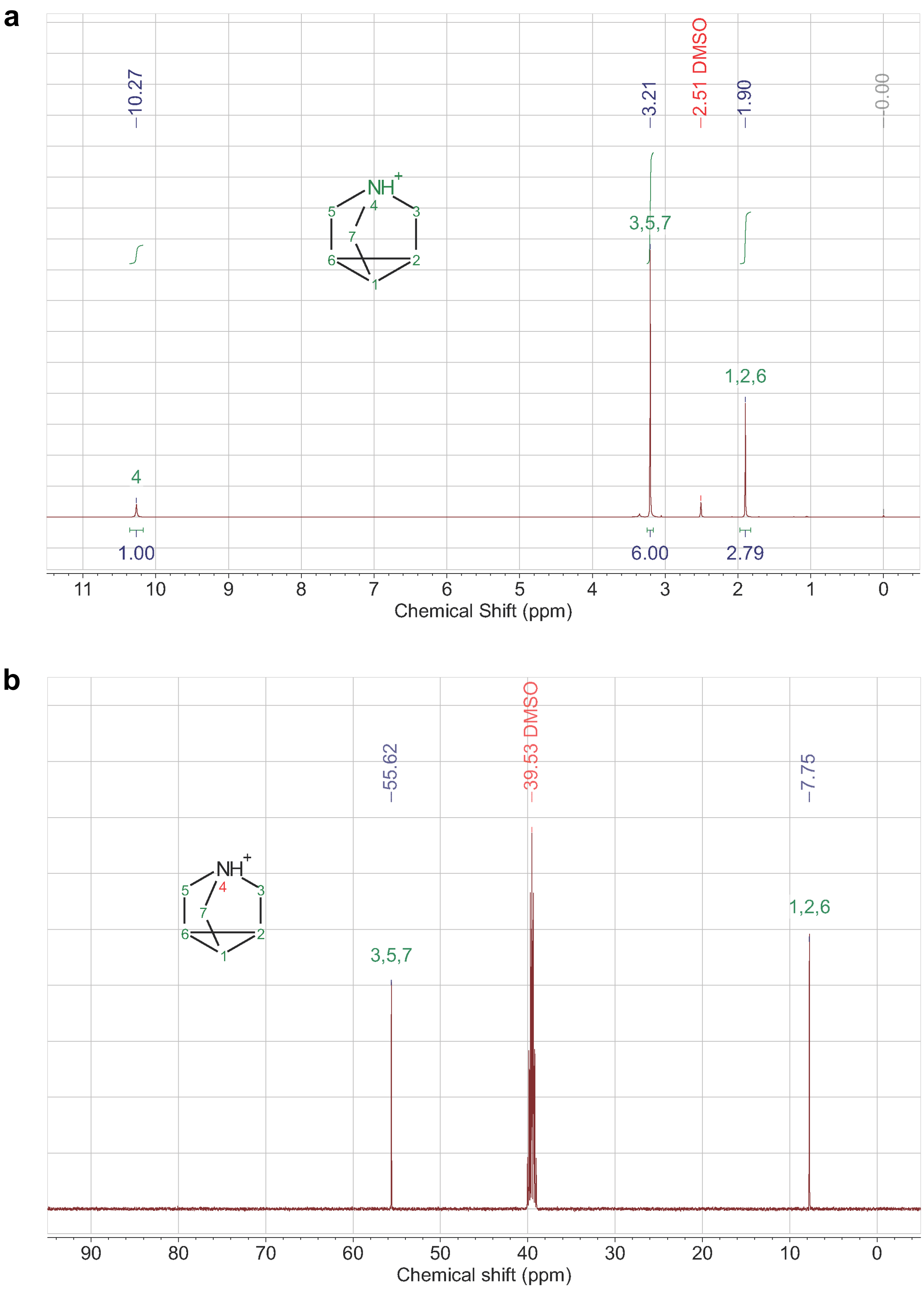

Supplementary Figure 2. (a) ^{1}H and (b) ^{13}C NMR spectra of 4-azatricyclo[2.2.1.0^{2,6}]-heptanium iodide in DMSO-*d*$_6$.

a

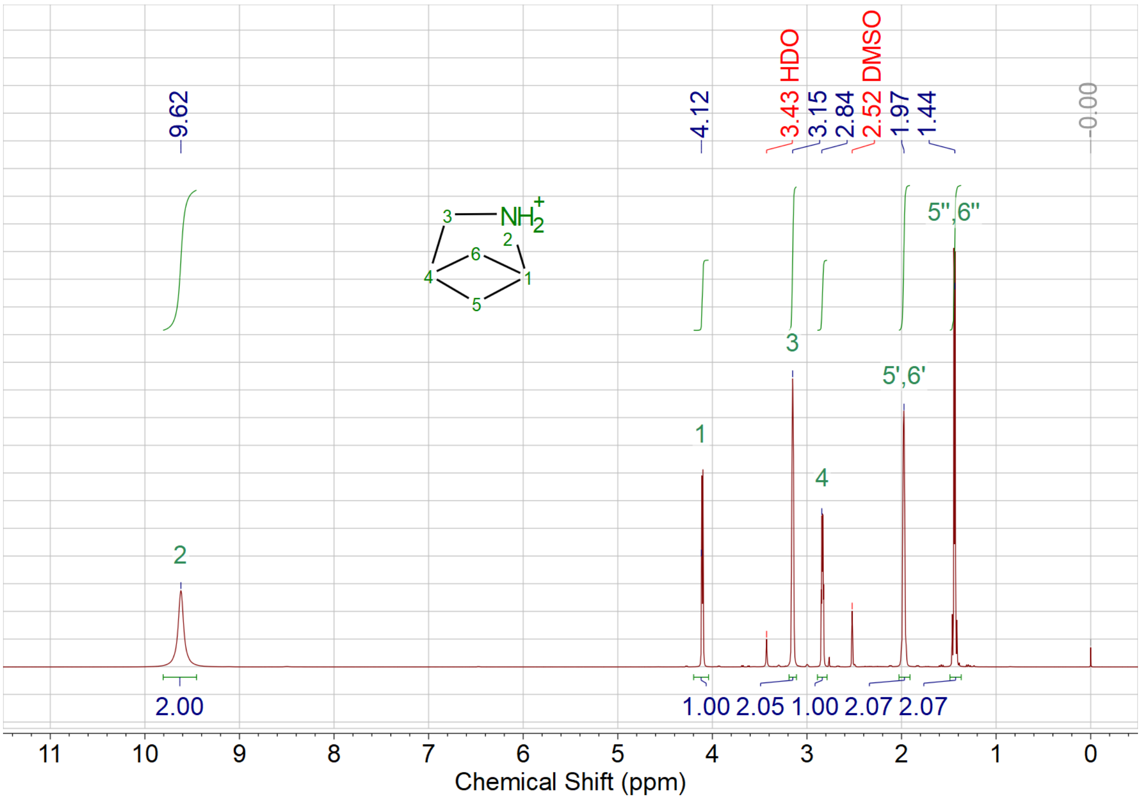

b

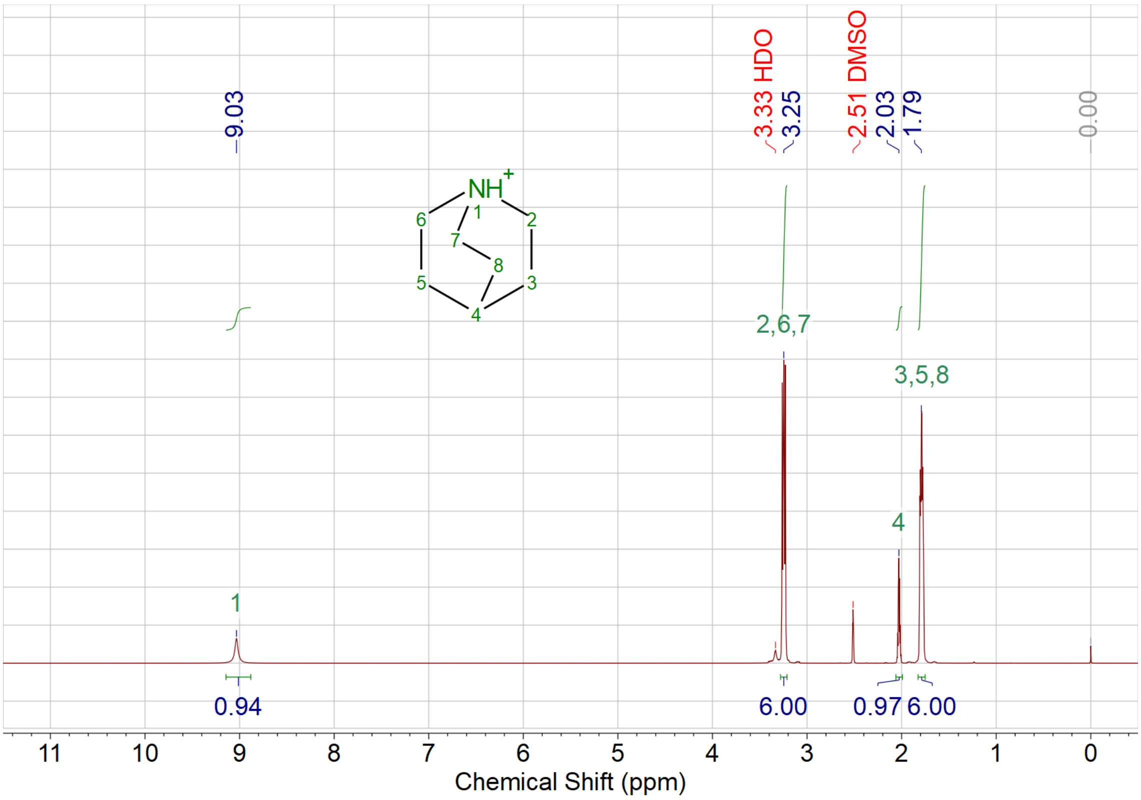

Supplementary Figure 3. ^{1}H NMR spectra of (a) 2-azabicyclo[2.1.1]hexanium and (b) quinuclidinium iodide in DMSO-d_6.

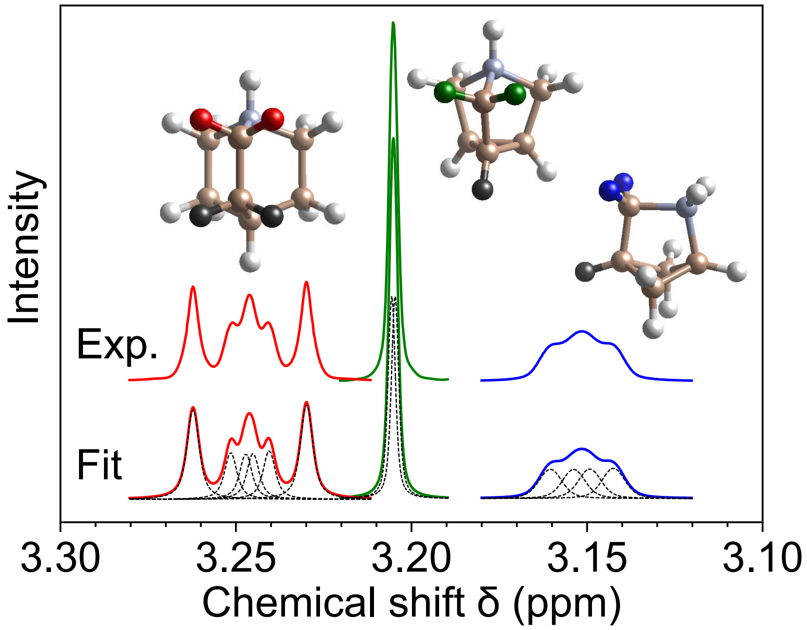

Supplementary Figure 4. NMR peak fitting for the α methylene protons of Abh^+ (blue), Ath^+ (green), and Q^+ (red). Top row: Experimental data. Bottom row: Fitted curves using identical Voigt lineshapes (black dash lines).

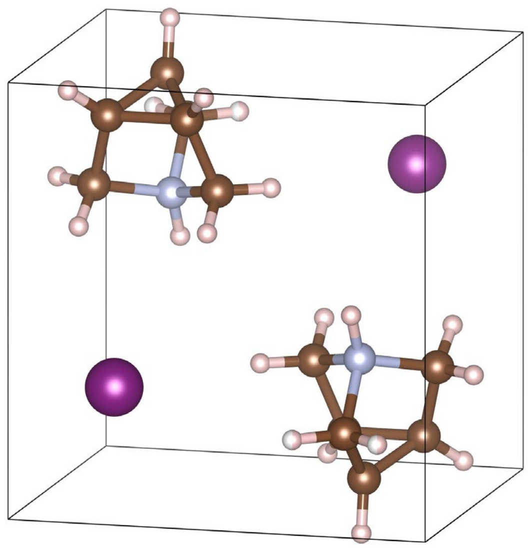

Supplementary Figure 5. Single-crystal structure of 4-azatricyclo[2.2.1.0^{2,6}]heptanium iodide (AthI) at 150 K. Brown, gray, pink, and purple spheres stand for C, N, H, and I, respectively.

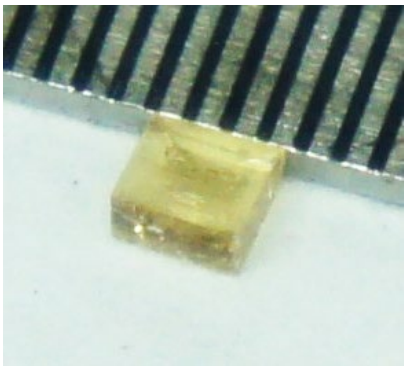

Supplementary Figure 6. Photograph of an AthMn(N_3)$_3$ single crystal next to 0.5 mm rulings.

Supplementary Table 1. Crystal data and structural refinement for AthI.

Formula	$C_6H_{10}NI$ (FW = 223.06)
CCDC number	2073935
Temperature (K)	150
Crystal system	Monoclinic
Space group	$P2_1/m$ (No. 11)
a (Å)	5.6422(4)
b (Å)	7.9766(5)
c (Å)	8.0323(6)
α, γ (°)	90
β (°)	94.695(2)
V (Å^3)	360.29(4)
Z	2
V/Z (Å^3)	180.15
ρ_{calc} (g·cm^{-3})	2.056
μ (mm^{-1})	4.344
F(000)	212.0
Radiation	Mo $K\alpha$ (λ = 0.71073 Å)
2θ range for data collection (°)	5.088 to 52.796
Index ranges	$-7 \le h \le 7$<br>$-9 \le k \le 9$<br>$-10 \le l \le 10$
Reflections collected	4910
Independent reflections	788 [R_{int} = 0.0297, R_{sigma} = 0.0195]
Data/restraints/parameters	788/0/43
Goodness-of-fit on F^2	1.138
Final R indexes [$I \ge 2\sigma(I)$]	R_1 = 0.0159, wR_2 = 0.0398
Final R indexes [all data]	R_1 = 0.0166, wR_2 = 0.0401
Largest diff. peak/hole (e·Å^{-3})	0.52/−0.59

Supplementary Table 2. Crystal data and structural refinement for $AthMn(N_3)_3$.

Formula	$C_6H_{10}N_{10}Mn$ (FW = 277.18)		
CCDC number	2073936	2073937	2073938
Temperature (K)	150	300	350
Crystal system	Cubic	Cubic	Cubic
Space group	$Pa\bar{3}$ (No. 205)	$Pa\bar{3}$ (No. 205)	$Pm\bar{3}m$ (No. 221)
$a = b = c$ (Å)	12.8542(4)	12.9235(4)	6.4934(9)
V (Å^3)	2123.9(2)	2158.4(2)	273.8(1)
Z	8	8	1
V/Z (Å^3)	265.49(3)	265.80(3)	273.8(1)
ρ_{calc} (g·cm^{-3})	1.734	1.706	1.620
μ (mm^{-1})	1.240	1.220	1.200
F(000)	1128.0	1128.0	131.0
Radiation	Mo $K\alpha$ (λ = 0.71073 Å)		
2θ range for data collection (°)	5.49 to 54.036	5.46 to 54.152	6.274 to 54.078
Index ranges	$-16 \le h \le 16$<br>$-16 \le k \le 16$<br>$-16 \le l \le 16$		$-8 \le h \le 8$<br>$-5 \le k \le 8$<br>$-7 \le l \le 8$
Reflections collected	26578	26879	1168
Independent reflections	790<br>[R_{int} =0.0771,<br>R_{sigma} = 0.0157]	800<br>[R_{int} = 0.0468,<br>R_{sigma} = 0.0110]	88<br>[R_{int} = 0.0269,<br>R_{sigma} = 0.0106]
Data/restraints/parameters	790/0/53	800/0/53	88/0/13
Goodness-of-fit on F^2	1.060	1.089	1.218
Final R indexes [$I \ge 2\sigma(I)$]	R_1 = 0.0290,<br>wR_2 = 0.0662	R_1 = 0.0326,<br>wR_2 = 0.0853	R_1 = 0.0418,<br>wR_2 = 0.1235
Final R indexes [all data]	R_1 = 0.0508,<br>wR_2 = 0.0769	R_1 = 0.0487,<br>wR_2 = 0.0966	R_1 = 0.0418,<br>wR_2 = 0.1235
Largest diff. peak/hole (e·Å^{-3})	0.23/−0.36	0.18/−0.22	0.41/−0.35

Supplementary Table 3. Crystal data and structural refinement for AbhMn(N_3)$_3$.

Formula	$C_5H_{10}N_{10}Mn$ (FW = 265.17)	
CCDC number	2073939	2073940
Temperature (K)	280	320
Crystal system	Orthorhombic	Cubic
Space group	*Pnma* (No. 62)	$Pm\bar{3}m$ (No. 221)
a (Å)	8.839(1)	6.3883(8)
b (Å)	12.692(2)	6.3883(8)
c (Å)	9.161(1)	6.3883(8)
α, β, γ (°)	90	90
V (Å^3)	1027.7(2)	260.7(1)
Z	4	1
V/*Z* (Å^3)	256.92(5)	260.7(1)
ρ_{calc} (g·cm^{-3})	1.714	1.689
μ (mm^{-1})	1.277	1.258
F(000)	540	135
Radiation	Mo *K*α (λ = 0.71073 Å)	
2θ range for data collection (°)	5.484 to 54.242	11.058 to 54.172
Index ranges	$-11 \le h \le 11$<br>$-16 \le k \le 16$<br>$-11 \le l \le 11$	$-8 \le h \le 7$<br>$-8 \le k \le 5$<br>$-8 \le l \le 8$
Reflections collected	14307	1718
Independent reflections	1178 [R_{int} = 0.0660, R_{sigma} = 0.0265]	82 [R_{int} = 0.0249, R_{sigma} = 0.0082]
Data/restraints/parameters	1178/1/85	82/0/11
Goodness-of-fit on F^2	1.175	1.283
Final *R* indexes [$I \ge 2\sigma(I)$]	R_1 = 0.0573, wR_2 = 0.1631	R_1 = 0.0354, wR_2 = 0.1000
Final *R* indexes [all data]	R_1 = 0.0717, wR_2 = 0.1754	R_1 = 0.0354, wR_2 = 0.1000
Largest diff. peak/hole (e·Å^{-3})	0.56/−0.48	0.33/−0.50

Supplementary Table 4. Crystal data and structural refinement for $QMn(N_3)_3$.

Formula	$C_7H_{14}N_{10}Mn$ (FW = 293.22)		
CCDC number	2073941	2073943	2073942
Temperature (K)	300	360	330 (cooling)
Crystal system	Cubic	Cubic	Tetragonal
Space group	$Pa\bar{3}$ (No. 205)	$Pm\bar{3}m$ (No. 221)	*P4/nmm* (No. 129)
a (Å)	13.1893(4)	6.641(1)	13.216(2)
b (Å)	13.1893(4)	6.641(1)	13.216(2)
c (Å)	13.1893(4)	6.641(1)	6.604(1)
V (Å^3)	2294.4(2)	292.8(2)	1153.5(4)
Z	8	1	4
V/Z (Å^3)	286.8(1)	292.8(2)	288.2(1)
ρ_{calc} (g·cm^{-3})	1.698	1.663	1.688
μ (mm^{-1})	1.152	1.129	1.146
F(000)	1208	151.0	604.0
Radiation	Mo $K\alpha$ (λ = 0.71073 Å)		
2θ range for data collection (°)	5.35 to 54.214	6.136 to 52.372	4.358 to 52.686
Index ranges	$-13 \leq h \leq 16$<br>$-16 \leq k \leq 16$<br>$-16 \leq l \leq 16$	$-8 \leq h \leq 8$<br>$-8 \leq k \leq 8$<br>$-8 \leq l \leq 6$	$-16 \leq h \leq 16$<br>$-16 \leq k \leq 12$<br>$-8 \leq l \leq 8$
Reflections collected	29366	1663	13005
Independent reflections	851<br>[R_{int} =0.0958, R_{sigma} = 0.0175]	84<br>[R_{int} = 0.0314, R_{sigma} = 0.0105]	678<br>[R_{int} = 0.1035, R_{sigma} = 0.0383]
Data/restraints/parameters	851/0/57	84/0/14	678/0/65
Goodness-of-fit on F^2	1.071	1.277	1.109
Final R indexes [$I \geq 2\sigma(I)$]	R_1 = 0.0315, wR_2 = 0.0576	R_1 = 0.0421, wR_2 = 0.1125	R_1 = 0.1034, wR_2 = 0.2096
Final R indexes [all data]	R_1 = 0.0618, wR_2 = 0.0696	R_1 = 0.0421, wR_2 = 0.1125	R_1 = 0.1433, wR_2 = 0.2320
Largest diff. peak/hole (e·Å^{-3})	0.22/−0.35	0.39/−0.27	0.75/−1.49

Crystallographic Information Files (CIFs)

CIF file	A-level alerts	B-level alerts
AthI_150K	No	No
AthMn(N3)3_150K	No	No
AthMn(N3)3_300K	No	No
AthMn(N3)3_350K	No	PLAT043, PLAT088, PLAT241
AbhMn(N3)3_280K	No	PLAT241, PLAT242
AbhMn(N3)3_320K	No	PLAT043, PLAT088, PLAT241, PLAT940
QMn(N3)3_300K	No	No
QMn(N3)3_330K	No	PLAT043, PLAT241, PLAT242
QMn(N3)3_360K	No	PLAT043, PLAT088, PLAT241, PLAT940

Explanation to the B-level alerts:

PLAT043_ALERT_1_B Calculated and Reported Mol. Weight Differ by .... ? Check

This difference between calculated and reported molecular weight is due to omitted hydrogens and the disordered A-site cation.

PLAT088_ALERT_3_B Poor Data / Parameter Ratio .... ? Note

The cubic phase yields only a few diffraction points, insufficient for resolving the disordered A-site cation. This index is inappropriate for highly symmetric but disordered structures.

PLAT241_ALERT_2_B High 'MainMol' Ueq as Compared to Neighbors of ....N003 Check

PLAT242_ALERT_2_B Low 'MainMol' Ueq as Compared to Neighbors of .... Mn01 Check

Disordered terminal N^- of the azide bridge was described by an oblate ellipsoid, but Mn^{2+} and the middle N have small and isotropic U_{eq} values.

PLAT0940_ALERT_3_B Fsqd Refinement With I > n * Sigma(I) Only .... Please Check

All the diffraction points have high intensity, so numbers of independent and $I > 2\sigma$ points are equal.

Supplementary References

1. Agrawal, K. C. *et al.* A new method for the synthesis of ethyl (1α, 5α, 6α)-3-benzyl-3-azabicyclo[3.1.0.]hexane-2,4-dione-6-carboxylate: An intermediate for the trovafloxacin side chain. *Indian J. Chem. B* **43**, 873-875 (2004).
2. Payne, G. B. Cyclopropanes from reactions of ethyl dimethylsulfuranylideneacetate with α,β-unsaturated compounds. *J. Org. Chem.* **32**, 3351-3355 (1967).
3. Conway, L. P. *et al.* The aqueous n-phosphorylation and n-thiophosphorylation of aminonucleosides. *RSC Adv.* **4**, 38663-38671 (2014).
