# Supplementary material for: Order–disorder transition of a rigid cage cation embedded in a cubic perovskite
Source: Nat Commun. 2021 Jun 10;12:3548. doi: 10.1038/s41467-021-23917-z (PMC8192939; doi:10.1038/s41467-021-23917-z)
Supplement: Supplementary file 12 — Description of additional supplementary files [file 41467_2021_23917_MOESM12_ESM.docx]

**Description of additional supplementary files**

Title: Dataset 1

Description: Crystallographic Information File for AthI at 150 K.

Title: Dataset 2

Description: Crystallographic Information File for AbhMn(N_3_)_3_ at 280 K.

Title: Dataset 3

Description: Crystallographic Information File for AbhMn(N_3_)_3_ at 320 K.

Title: Dataset 4

Description: Crystallographic Information File for AthMn(N_3_)_3_ at 150 K.

Title: Dataset 5

Description: Crystallographic Information File for AthMn(N_3_)_3_ at 300 K.

Title: Dataset 6

Description: Crystallographic Information File for AthMn(N_3_)_3_ at 350 K.

Title: Dataset 7

Description: Crystallographic Information File for QMn(N_3_)_3_ at 300 K.

Title: Dataset 8

Description: Crystallographic Information File for QMn(N_3_)_3_ at 330 K.

Title: Dataset 9

Description: Crystallographic Information File for QMn(N_3_)_3_ at 360 K.
